# Single-Walled Carbon Nanotube-Germanium Heterojunction for High-Performance Near-Infrared Photodetector

**DOI:** 10.3390/nano12081258

**Published:** 2022-04-08

**Authors:** Tao Qi, Yaolun Yu, Yanyan Hu, Kangjie Li, Nan Guo, Yi Jia

**Affiliations:** 1 Department of Communication Engineering, Nanjing University of Science and Technology, Nanjing 210094, China; qt18101084039@163.com; 2 Qian Xuesen Laboratory of Space Technology, China Academy of Space Technology, Beijing 100094, China; yuyaolun@qxslab.cn (Y.Y.); 18811791066@163.com (Y.H.); lkjzqt1900406120@163.com (K.L.)

**Keywords:** SWCNT-Ge heterojunction, near-IR photodetector, ozone treatment

## Abstract

In this research, we report on a high-performance near-infrared (near-IR) photodetector based on single-walled carbon nanotube-germanium (SWCNT-Ge) heterojunction by assembling SWCNT films onto n-type Ge substrate with ozone treatment. The ozone doping enhances the conductivity of carbon nanotube films and the formed interfacial oxide layer (GeO_x_) suppresses the leakage current and carriers’ recombination. The responsivity and detectivity in the near-IR region are estimated to be 362 mA W^−1^ and 7.22 × 10^11^ cm Hz^1/2^ W^−1^, respectively, which are three times the value of the untreated device. Moreover, a rapid response time of ~11 μs is obtained simultaneously. These results suggest that the simple SWCNT-Ge structure and ozone treatment method might be utilized to fabricate high-performance and low-cost near-IR photodetectors.

## 1. Introduction

High-performance infrared detectors are widely studied for many important applications [1,2,3,4,5,6,7,8], such as industrial, security, environmental monitoring, and space exploration. In the past decades, many efforts have been made to develop infrared detectors with narrow bandgap semiconductors, such as HgCdTe [9], InSb [10], PbS [11], PbTe [12], PtSe_2_ [13] and HgTe [14]. Although those devices have excellent performance, their constituent materials often have highly toxic heavy metal elements, such as Hg, Cd, and Pb. In addition, the fabrication process of these devices typically requires complex instruments, which in turn increases the price of those devices. Therefore, these shortcomings become the major obstacles for their widespread application.

Carbon nanotubes, with their unique one-dimensional structure, excellent physical and photoelectric properties, tunable bandgap, spectral absorption ranging from visible to infrared light, are promising photodetector materials [15,16,17,18,19]. Many CNT-based optoelectronic devices have been developed in recent years, including solar cells [20], infrared detectors [21,22,23] and ultraviolet detectors [24]. In particular, in the field of infrared detectors, CNT bolometers [25,26,27,28], CNT photoconductors [29] and CNT photodiodes [30,31,32,33] have been investigated. Moreover, CNT films can be constructed with various semiconductors to form heterojunctions for infrared light detection. For example, Ong et al. [34] utilized multi-walled carbon nanotube films to fabricate the Schottky junction with a silicon wafer, showing a detecting capability in the range of 8–12 μm with a fast response time (16 ms). Zhang et al. [35] fabricated a nano-photodetector by forming a carbon nanotube film-graphene Schottky heterojunction. The as-fabricated photodetector exhibited high responsivity (209 mA W^−1^), detectivity (4.87 × 10^10^ cmHz^1/2^ W^−1^) and fast response speed (68 μs) in a wide spectrum range. However, these heterojunctions usually suffer from relatively large leakage currents and surface recombination, which become a key obstacle limiting the device performance. Therefore, it is necessary to find solutions for obtaining higher responsivity together with lower dark currents for high-performance infrared detectors.

In this work, we report that SWCNT-germanium (SWCNT-Ge) heterojunctions prepared by a simple transfer and ozone treatment technique exhibit high photoresponse in the near-IR region. In this structure, Ge with its bandgap of 0.66 eV, plays an important role in spectral absorption; SWCNT films with their high conductivity and optical transmittance, serve as a highly conductive network for charge transport and a transparent window for light illumination; ozone molecules are introduced to improve the conductivity of SWCNT films while forming an oxide layer at the heterojunction interface, which can greatly suppress the leakage current and surface recombination. As a result, the responsivity, detectivity, and rise/fall response time of the ozone-treated SWCNT-Ge photodetector under 1310 nm light illumination are 362 mA W^−1^ and 7.22 × 10^11^ cm Hz^1/2^ W^−1^ and 9 μs/11 μs, respectively. These results show that the SWCNT-Ge heterojunction will have great potential for use as a near-IR detector in communication and imaging.

## 2. Materials and Methods

### 2.1. SWCNT Films Synthesis and Treatment

The SWCNT films were synthesized by a floating catalyst-chemical vapor deposition (CVD) process in a quartz tube in a horizontal furnace, with xylene as the carbon source and ferrocene/sulfur as catalyst precursor. Solutions of the carbon source and catalyst were injected into the quartz tube by a precision pump (30 μL/min) and then carried by the gases (H_2_ ~400 sccm and Ar ~2000 sccm) into the high-temperature reaction zone (1150~1170 °C). The as-produced SWCNT films were blown by the carrier gas and collected downstream of the quartz tube.

The collected SWCNT films were sequentially immersed into H_2_O_2_ (30 wt.%) and HCl (36.5 wt.%) to remove the amorphous carbon and Fe catalyst residues then rinsed with deionized water until the pH reached neutral. By adding a few drops of ethanol, the purified CNT films fully expanded into uniform thin films on the water surface for further assembling the heterojunction device.

### 2.2. Device Assembly

N-type Ge wafers with a resistivity of 2~4 Ω·cm were firstly immersed into dilute HF solution to remove the surface oxide layer, then washed with DI water and further dried by nitrogen gas. A black insulating tape with a circular hole (5 mm in diameter) was attached to the Ge wafer. Then, the floating SWCNT films were slowly lifted onto the Ge wafer to form the SWCNT-Ge heterojunction. The upper and lower electrodes were extracted from the SWCNT films and Ge wafer by silver paste and indium gallium alloy to form the ohmic contacts.

### 2.3. Characterization

The morphology of the SWCNT films was observed by scanning electron microscope (SEM, Helios G4 CX, FEI, Hillsboro, OR, USA). Raman spectra were measured using the Renishaw Invia with a 532 nm laser source. The photoelectric response tests of the devices were carried out in a Lake Shore probe station with a 1310 nm laser as the light source, and the laser powers were calibrated by the optical power meter (PM100D, Thorlabs, Newton, NJ, USA). The *I*-*V* characteristics of the devices were measured by a semiconductor device analyzer (B1500A, Keysight, Santa Rosa, CA, USA). The response speed was obtained via a digital oscilloscope (MSOX3024T, Keysight, Santa Rosa, CA, USA). In the measurement of spectral response, the DTGS detector of the infrared spectrometer (VERTEX 80V, Bruker, Billerica, MA, USA) was replaced by our device. The evolution of the oxide layer on the Ge wafer surface during the ozone treatment was measured by depth-resolved Auger electron spectra (PHI-700, ULVAC-PHI, Chigasaki, Japan) with an Argon ion etching rate of 1 nm/min (for SiO_2_).

## 3. Results

### 3.1. Characterization of the SWCNT-Ge Heterojunction Photodetectors

**Figure 1:**
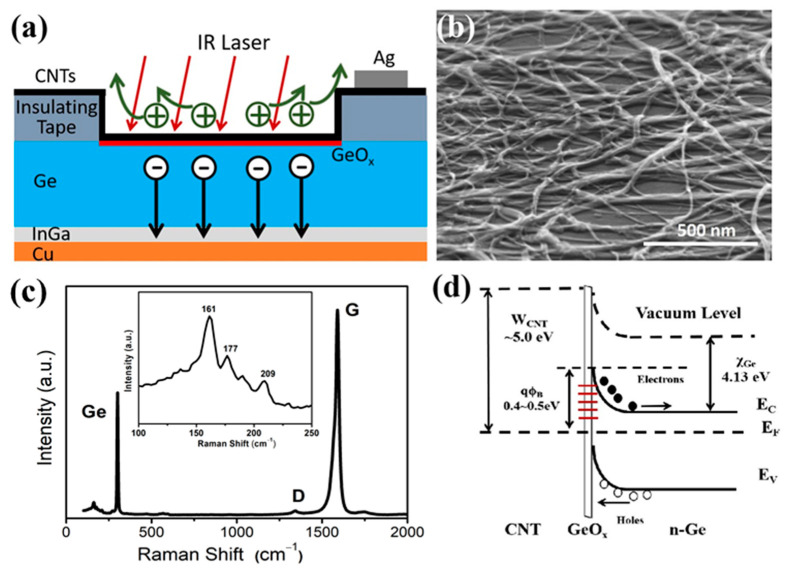
The SWCNT-Ge heterojunction photodetector. (**a**) A schematic illustration of the SWCNT-Ge photodetector; (**b**) SEM image of the SWCNT-Ge interface; (**c**) Raman spectra of the SWCNT-Ge photodetector; (**d**) Energy band diagram of the SWCNT-Ge photodetector.

Figure 1a shows the schematic diagram of the SWCNT-Ge near-IR photodetector. SWCNT films were uniformly transferred on the Ge substrate to form a heterojunction with an area of ~0.2 cm^2^, while the surrounding parts of the SWCNT films were led out as an electrode through the silver paste. Figure 1b is an SEM image of the SWCNT-Ge interface morphology, which shows that the SWCNT films have a network structure with very few impurities and constitute good contact with the Ge substrate. The Raman spectrum of the SWCNT-Ge heterojunction (shown in Figure 1c) mainly consists of a peak for Ge (~320 cm^−1^), an extremely low D peak (~1360 cm^−1^) and a very strong G peak (~1580 cm^−1^) for CNT, while the ratio of *I*_G_/*I*_D_ exceeds 50, indicating that the SWCNT films have excellent crystallization and very little amorphous carbon impurities. Three peaks (161 cm^−1^, 177 cm^−1^, and 209 cm^−1^) in the radial breathing mode region correspond to the CNT tube diameters of 1.39 nm, 1.27 nm and 1.07 nm, respectively. Figure 1d illustrates the energy band diagram of the SWCNT-Ge photodetector. Due to the strong Fermi-level pinning effect with charge neutrality level close to the valence band edge of n-Ge, there is a high Schottky barrier height (q*Φ*b, 0.4~0.5 eV) [36,37], which can separate and drive photogenerated holes and electrons to SWCNT films and Ge substrate, respectively. With ozone treatment, an oxide layer (GeO_x_) is formed at the interface, which greatly suppresses the leakage current and surface recombination.

### 3.2. Performances of the As-Prepared SWCNT-Ge Heterojunction Photodetectors

**Figure 2:**
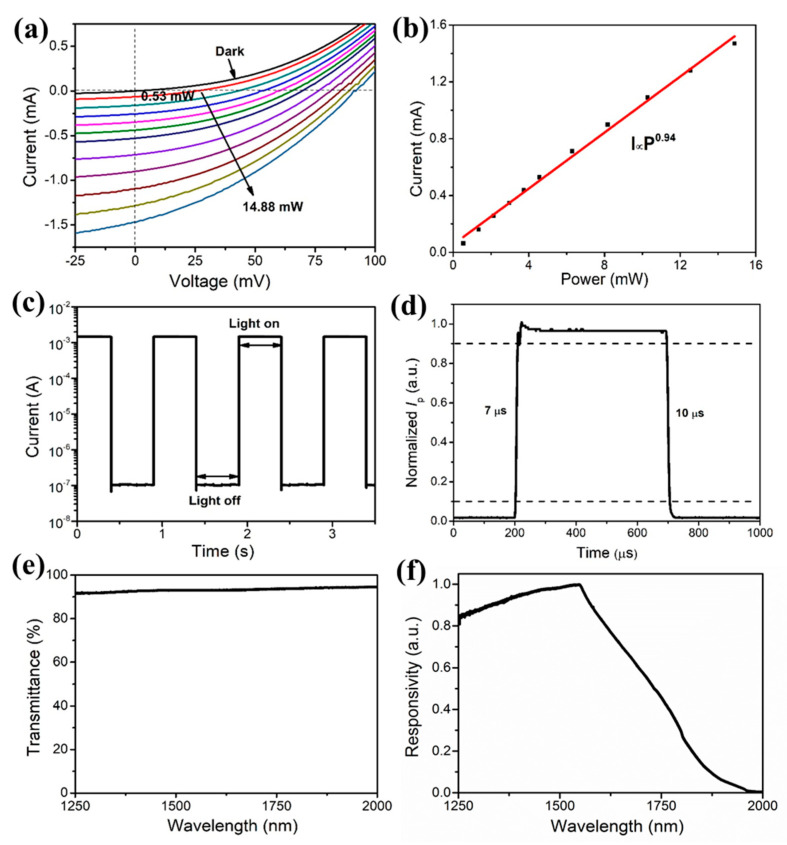
Performances of the as-prepared SWCNT-Ge photodetector under near-IR laser illumination. (**a**) *I*-*V* curves of the SWCNT-Ge photodetector with different laser powers illumination (dark, 0.53 mW, 1.34 mW, 2.14 mW, 2.96 mW, 3.73 mW, 4.56 mW, 6.29 mW, 8.17 mW, 10.27 mW, 12.55 mW and 14.88 mW, respectively); (**b**) The relationship between photocurrent and laser power; (**c**) Response characteristics of the SWCNT-Ge photodetector under pulsed laser illumination (1 Hz) at 0 V; (**d**) A normalized cycle measurement at 1 kHz for estimating the response and recovery time (7/10 μs); (**e**) The transmittance of SWCNT films from 1250 to 2000 nm; (**f**) Spectral response of the SWCNT-Ge photodetector.

In order to study the effects of ozone treatment on the device performance, the photo-response characteristics of as-prepared SWCNT-Ge heterojunction photodetectors without ozone treatment were investigated first. Figure 2a is the *I*-*V* curves of an as-prepared device tested under both dark and light (with the laser powers (*P*_opt_) from 0.53 mW to 14.88 mW) conditions at room temperature. The detection performances were characterized by illuminating the device with a calibrated irradiation intensity of 0.53 mW 1310 nm IR laser. The light *I*-*V* curve shows an open-circuit voltage (*V*_oc_) of 25 mV and a photocurrent (*I*_p_, at 0 V) of 0.065 mA. The responsivity (*R*) and detectivity (D*) of the photodetector are calculated by [38,39,40]:

*R* = *I*_p_/*P*_opt_ (1)

D* = A^1/2^ *R*/(2q*I*_d_)^1/2^ = A^1/2^(*I*_p_/*P*_opt_)/(2q*I*_d_)^1/2^ (2)

where *I*_p_, *P*_opt_, q, A and *I*_d_ are the photocurrent, the incident laser power, the elementary charge (1.6 × 10^−19^ C), the active detection area of the photodetector (~0.2 cm^2^ in this paper) and the dark current, respectively.

From the above formulas, the *R* and D* of the device at zero bias are calculated to be 123 mA W^−1^ and 2.90 × 10^11^ cm Hz^1/2^ W^−1^, respectively.

In order to further reveal the response characteristics of the SWCNT-Ge heterojunction, the dependence between the laser power and the photocurrent was investigated. When the laser power increases from 0.53 to 14.88 mW, the photocurrent (at 0 V) increases from 0.065 to 1.48 mA. The relationship between photocurrent and laser power has been summarized in Figure 2b and can be fitted by:

*I*_p_∝*P*_opt_*^θ^* (3)

where *θ* is an empirical value related to the recombination process of excited carriers. The fitting result shows that *θ* = 0.94, indicating that the photocurrent is almost linearly related to the laser power.

Further, the transient response characteristics of the SWCNT-Ge heterojunction were carried out under 1 Hz light pulsed illumination at zero bias (Figure 2c). With the laser switching on and off alternately, the photocurrent and dark current of the device is 1.48 × 10^−3^ A and 1.1 × 10^−7^ A, respectively. The ratio between the photocurrent and dark current is about 1.35 × 10^4^, revealing that the device has good photoelectric characteristics and exhibits stable reversibility. Figure 2d shows a single cycle of the photo-response under 1 kHz light illumination. An oscilloscope was used to monitor the time dependence of the current. The rise time defined as the time interval for the current rising from 10% to 90% of its peak value is 7 μs, and the recovery time defined reversely is 10 μs. This response speed is much quicker than most nanomaterials-based photodetectors [41,42,43].

Figure 2e shows the spectral transmittance curves of the SWCNT films, and the SWCNT films with a porous structure achieve a transmission rate of more than 90% in the spectral range from 1250 nm to 2000 nm. The porous structure and high light transmission of CNT films increase the number of photons reaching the underlying heterojunction region, resulting in a high photoresponse. Figure 2f shows the normalized photocurrent spectrum of the SWCNT-Ge heterojunction photodetector. The maximum quantum efficiency occurs at the wavelength of 1550 nm. When the wavelength is longer than 1900 nm (close to the bandgap of Ge), the quantum efficiency of the device is almost 0, indicating that the photoresponse here mainly comes from the absorption of incident photons in the Ge substrate to generate the photocarriers.

### 3.3. Enhancing the Performances of the SWCNT-Ge Heterojunction Photodetectors by Ozone Treatment

**Figure 3:**
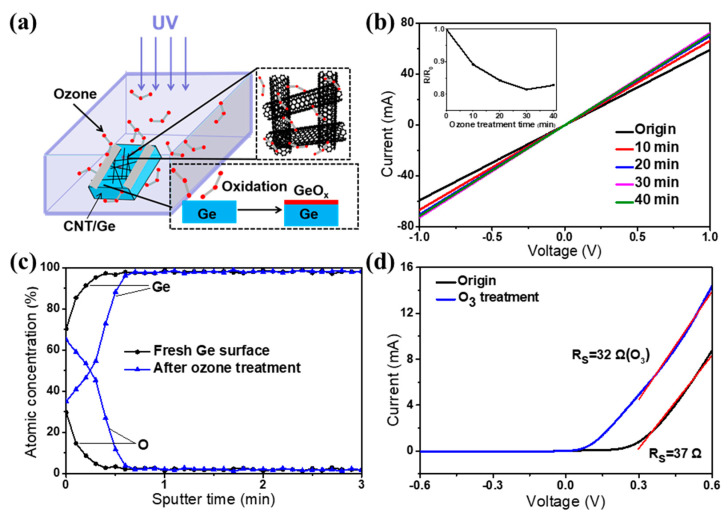
Ozone doping process and characteristics of SWCNT-Ge photodetector. (**a**) A schematic illustration of ozone doping process at the SWCNT films and Ge interface; (**b**) *I*-*V* curves of the SWCNT films with different ozone treatment times. Inset shows the relationship between the sheet resistances of SWCNT films and ozone treatment time; (**c**) Depth-resolved Auger electron spectrum of germanium and oxygen atomic concentrations in a fresh Ge substrate and after ozone treatment; (**d**) Dark *I*-*V* curves of the SWCNT-Ge photodetector before and after ozone treatment.

In general, the interface state plays a critical role in the performance of heterojunction optoelectronic devices. Here, we used the UV light-generated ozone molecules to treat the SWCNT-Ge heterojunction interface at different times, as shown in the schematic diagram of Figure 3a. First, the large specific surface area of the SWCNT films allows the ozone molecules to be effectively adsorbed and doped onto the SWCNT surface, thus increasing the carrier concentration of the SWCNT films. As a result, the sheet resistance of the SWCNT films was reduced by 18.5% after 30 min of ozone treatment (Figure 3b). Second, the porous structures of the SWCNT films allow the ozone molecules to reach the surface of the Ge substrate, thereby forming a thin oxide layer (GeO_x_). The evolution of GeO_x_ during the ozone treatment was analyzed by the depth-resolved Auger electron spectrum (Figure 3c). It shows that the oxygen concentration on the treated surface is significantly higher than that of the fresh sample, indicating that a thin oxide layer (~1 nm) was formed on the Ge substrate.

Benefiting from the reduced resistance of the SWCNT film and the formation of an interfacial oxide layer, the ozone treatment process has turned the original SWCNT-Ge structure into an MIS configuration (Figure 3a). The presence of the oxide layer can significantly suppress the leakage current and carrier recombination at the interface. As a result, in the dark *I*-*V* curves of Figure 3d, the series resistance is reduced by 13.5% (from 37 Ω to 32 Ω). To investigate the reproducibility of series resistance on different devices, 12 samples were assembled for ozone treatment and testing. Results show that the series resistances of ozone-treated devices decrease in the range of 10.3% to 21.3%.

**Figure 4:**
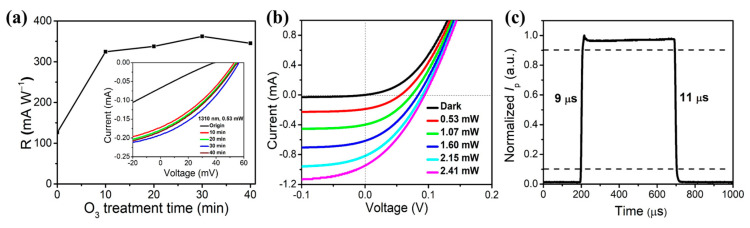
Performances of the ozone-treated SWCNT-Ge photodetector under the near-IR laser illumination. (**a**) Responsivity of SWCNT-Ge photodetector with different ozone treatment times, showing the maximum responsivity with 30 min treatment. The inset shows the *I*-*V* curves of the device with different ozone treatment times under laser illumination (1310 nm, 0.53 mW). (**b**) *I*-*V* curves of the ozone-treated SWCNT-Ge photodetector under different laser powers illumination; (**c**) A normalized cycle measurement at 1 kHz to estimate the response and recovery time.

Figure 4a shows the responsivity of an SWCNT-Ge photodetector with different ozone treatment times. It is found that the photocurrent and responsivity increased with increasing treatment time from 0 to 30 min. With 30 min ozone treatment, the photocurrent, *R* and D* achieve their maximum values of 0.192 mA, 362 mA W^−1^ and 7.22 × 10^11^ cm Hz^1/2^ W^−1^, respectively, which are about three times their initial values. These results are also much higher than the performances of a device with natural oxide layer without ozone treatment (with responsivity and detectivity are 49 mA W^−1^ and 1.3 × 10^11^ cm Hz^1/2^ W^−1^, respectively). The light power dependence of the device *I*-V curves was further characterized in Figure 4b. Compared with the untreated device, it can be seen that higher photocurrents can be obtained with low power illumination. This is because the thin oxide layer suppresses the surface recombination. More photocarriers migrate through the built-in electric field with little loss. In addition, the enhanced conductivity of SWCNT also accelerates the collection of carriers, leading to a high photocurrent. Figure 4c gives a single cycle photoresponse of the ozone-treated device under 1 kHz laser illumination at 0 V, showing a fast rise time and recovery time of 9 μs and 11 μs, respectively. The performance of the device could be further maintained by a suitable packaging method, for example, PDMS encapsulation [44]. Table 1 summarizes the reported CNT-Ge heterojunction and other similarly structured photodetectors as well as commercial Ge photodetectors. Overall, the device in our work has a relatively fast response speed and comparable responsivity and detectivity.

## 4. Conclusions

To summarize, we demonstrated a high-performance SWCNT-Ge photodetector by coating the SWCNT film onto the Ge substrate with ozone treatment. The ozone doping not only improved the conductivity of the SWCNT films by reducing the device’s series resistance but also produced an oxide layer at the SWCNT-Ge interface to suppress the charge carriers’ recombination. With 30-min ozone treatment, the responsivity and detectivity of the as-prepared device are estimated to be 362 mA W^−1^ and 7.22 × 10^11^ cm Hz^1/2^ W^−1^, respectively. This simple SWCNT-Ge structure and ozone treatment method may provide a new way for the development of future near-IR detectors.

## Figures and Tables

**Table 1:** Summary of the device performances of the CNT-Ge heterojunction photodetector and other similarly structured photodetectors.

Device Structure	Wavelength (nm)	Responsivity (mA W^−1^)	Rise Time (μs)	Fall Time (μs)	Detectivity (cm Hz^1/2^ W^−1^)	Ref.
CNT/Ge	1310	362	9	11	7.22 × 10^11^	this work
Gr/Ge	1550	51.8	23	108	1.38 × 10^10^	[41]
SWCNT/GaAs	780	274	1410	270	7.6 × 10^12^	[42]
MoTe_2_/GeO_2_/Ge	915	15.6	5000	5000	4.86 × 10^11^	[43]
Perovskite/SWCNT	1080	27	7.24	14.47	1.2 × 10^12^	[45]
SWCNT/C_60_	1200	97,500	3500	3500	1.17 × 10^9^	[46]
Gr/Al_2_O_3_/Ge	1550	1200			1.8 × 10^10^	[47]
Commercial Ge photodetector	1550				~2 × 10^11^	

## Data Availability

The data presented in this study are available on request from the corresponding author.
